# RESPECT TO HISTORY: VON PRESSER SYNDROME

**DOI:** 10.1590/0102-6720201700010001

**Published:** 2017

**Authors:** Alcino Lázaro da SILVA

**Affiliations:** Professor Emérito da Universidade Federal de Minas Gerais, Belo Horizonte, MG, Brasil. Ex-Presidente do Colégio Brasileiro de Cirurgia Digestiva

*"Visceral dilation, gastric ulcer at incisura angularis and normal pylorus: von Presser syndrome"*

Alcino Lázaro da Silva 

Working with the chloridropeptic ulcer, both duodenal and gastric ulcers, I would say that we have gained considerable experience. Over the years, we must not forget that everything happened in the pre-blocking stage of acid secretion era of the stomach; so, the chapter of chloridropptic ulcers today is mostly clinical.

Occasionally we found a clinical and surgical picture with gastrectase, gastric ulcer and pyloric stenosis. During the operation we observed visceral dilation, gastric ulcer located at the level of the incisura angularis and, surprisingly, normal pylorus, despite the radiography, suggesting stenosis.

**FIGURE 1:**
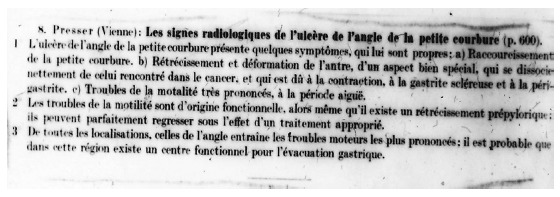
Original text from von Presser paper in1937

We did not find in the literature a similar picture, except the description of von Presser, K. in 1937^10^ from which we extracted what is read in Figure 1, and we call it "von Presser Syndrome".

**FIGURE 2:**
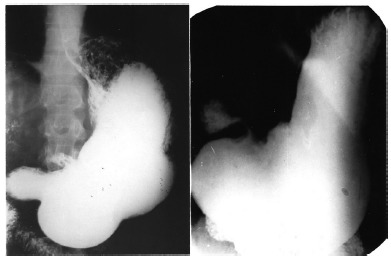
Two cases of von Presser syndrome where visceral dilatation, gastric ulcer at incisura angularis and normal pylorus are seen

Throughout our experience, we were able to operate 51 patients with gastric ulcer in the minor curvature at the level of the incisura angularis shown in Figure 2.

After the propedeutic was completed, the patients were submitted to the operation of choice following the standardization we performed. It is important to remember that all were treated before 1980, and at that time, the era of acid secretion blockers, the resurgence of *Helicobacter pylori*, and the use of antibiotics were also beginning.

All were submitted to partial antrectomy, vagotomy and gastroduodenal anastomosis in the small curvature.

In the surgical procedure gastrectase was confirmed and partial resection of the antrum showed the ulcer and, beside it, normal pylorus. In the operation it was demonstrated pyloric patency and not affected by the inflammatory process^2^ ^,^ ^3^ ^,^ ^4^ ^,^ ^5^ ^,^ ^6^ ^,^ ^7^ ^,^ ^8^ ^,^ ^9^ ^,^ ^11^ ^,^ ^12^

It is known that the stomach presents autonomous movements after the vagotomy. Their normalization is done up to three years. The origin of the new motor command is controversial, suggesting the existence of a pacemaker in the great or small curvature, or the origin and propagation of the movements by myogenic or neurogenic nature.

The operative techniques for the treatment of chloridropetic ulcer are numerous. In order to treat and prevent the sequelae of the operation, Resende Alves et al.^11^ ^,^ ^12^ proposed vagotomy, antrectomy and gastroduodenal anastomosis in the small curvature.

This was possible because the new knowledge of gastric physiology of that time allowed safely the vagotomy and the antrectomy as fundamental times in the prevention of the postoperative ulcer. This association would protect more, with less gastric resection and less mutilation. Alvarez^1^ tried to emphasize the existence of a region situated in the small curvature responsible for the gastric motor command, but his papers were forgotten.

In 1937, von Presser described a syndrome whose shortening and rectification of small curvature, associated with the difficulty of gastric emptying with normal pylorus, resulted in ulcers at the level of incisura angularis^10^.

As gastric emptying is done at the expense of harmonic movement and exceptionally by gravity, the conservation of a possible gastric motor zone - represented by the small curvature - would provide effective treatment without major impairment of stomach functions.

The small curvature, representing the primitive gastric tube, has the greater function of facilitating or conducting gastric emptying. It does so because it presents peculiar conditions in the embryological, anatomical, microscopic, physiological and pathophysiological aspects.

In the physiological baths of isolated organs, by studying the spontaneous movements of fragments of gastric regions in the dog, it was verified that in the proximal third of the small curvature there is a particular movement of the tonic type, with graphic representation that identifies it as gastric wave of type III.

The experience obtained with the clinical material under study seems to demonstrate that the restoration of the gastroduodenal transit at the level of the small curvature, protected by the vagotomy, offers good clinical results late and a lower rate of ulcer recurrence, thanks to gastric emptying near the normal one.

In conclusion, what can be said is that the small curvature allows the free passage of the intake and participates or triggers the control of the movements of the stomach. Is there a pacemaker? The unit is what facilitates the anti-clockwise movement during the malaxation of the food bolus? It is still not clarified what happens, functionally, when the incisura angularis is affected by the chloridropeptic ulcer.

At the time when there were no acid secretion blockers in the stomach, gastric surgery was iatrogenic or at high risk, and because *Helicobacter pylori* had not yet been described, many patients grew ulcers for years. With this, the gastric ulcer remained, evolved into a chronic ulcerated process and, when located in the incisura angularis, generated gastric stasis that participated in the syndrome that we call "von Presser"
